# Supplementary material for: Differential regulation of mRNA stability modulates transcriptional memory and facilitates environmental adaptation
Source: Nat Commun. 2023 Feb 17;14:910. doi: 10.1038/s41467-023-36586-x (PMC9936472; doi:10.1038/s41467-023-36586-x)
Supplement: Supplementary file 1 — Supplementary Information [file 41467_2023_36586_MOESM1_ESM.pdf]

# Supplementary material

## Differential regulation of mRNA stability modulates transcriptional memory and facilitates environmental adaptation

Bingnan Li[1,2], Patrice Zeis[3,†], Yujie Zhang[2], Alisa Alekseenko[2], Eliska Fürst[4], Yerma Pareja Sanchez[2], Gen Lin[3,‡], Manu M Tekkedil[3], Ilaria Piazza[4], Lars M. Steinmetz[3,5,6], Vicent Pelechano[2*]

Supplementary Figure 1

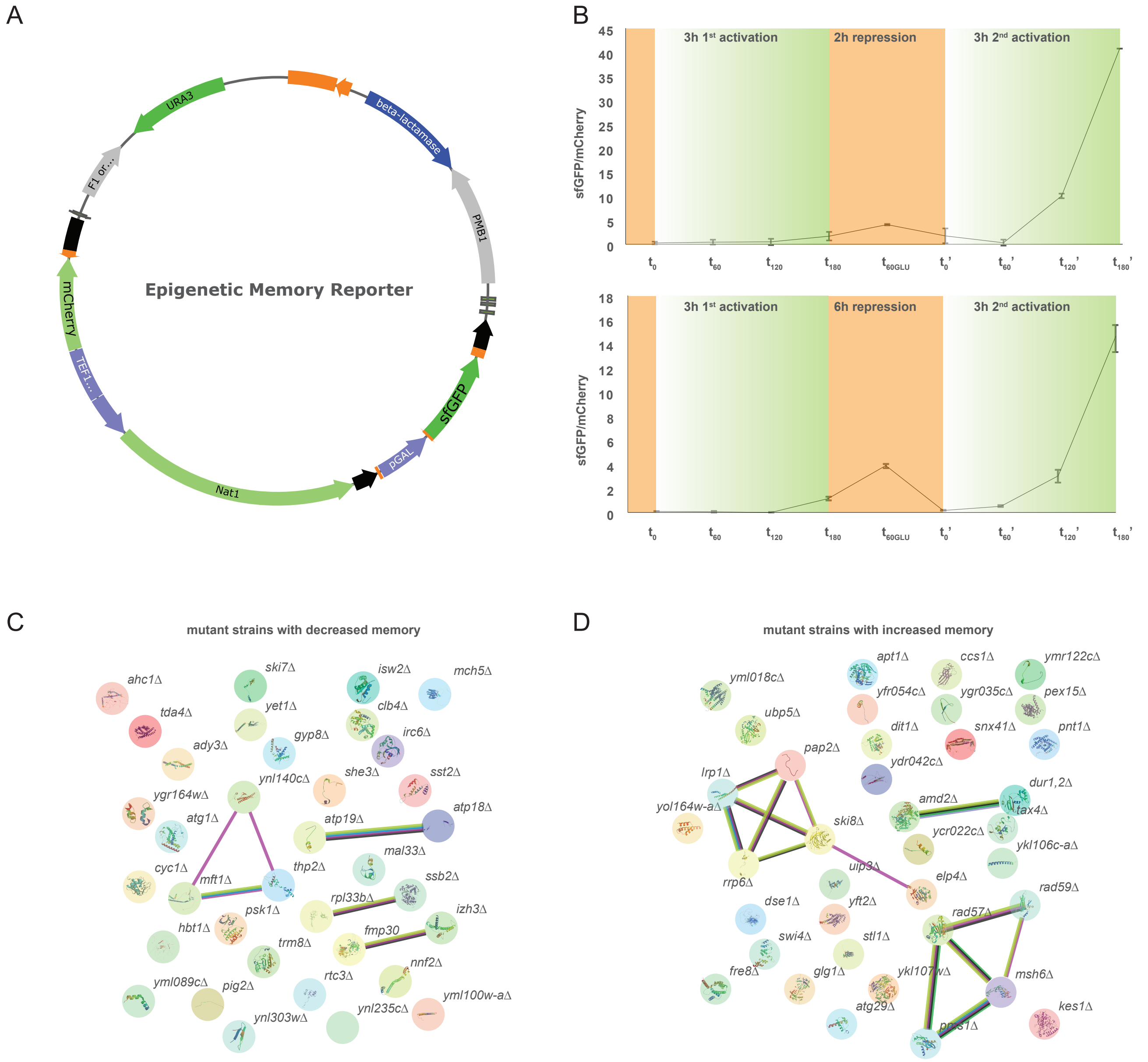


**Supplementary Figure 1: Extended information for transcriptional memory screen. A,** Details of the plasmid used as reporter for transcriptional memory using p416 TEF as backbone[43] . **B**, Plate reader measurements of the relative accumulation of *pGAL1-sfGFP* in relation to the constitutively expressed pTEF1-mCherry in a wild-type strain, confirming the ability of the used reporter to display transcriptional memory. Tests using both 2 hours (up) and 6 hours of repression in YPD (down) and 2 transformed clones are shown. As expected, protein accumulation is delayed in respect to transcriptional response. **C**, Network analysis for the 35 candidate genes putatively decreased transcriptional memory (Supplementary Data 1) using STRING v11.5[48]. blue indicate from curated databases, pink indicate experimentally determined, green indicate predicted interaction as gene neighborhood, light green indicate source as text mining, black indicate co-expression. **D**, Network analysis candidate genes putatively enhanced transcriptional memory (Supplementary Data 1) using STRING v11.5[48]. Colour code as in Supplementary Figure 1C.

Supplementary Figure 2

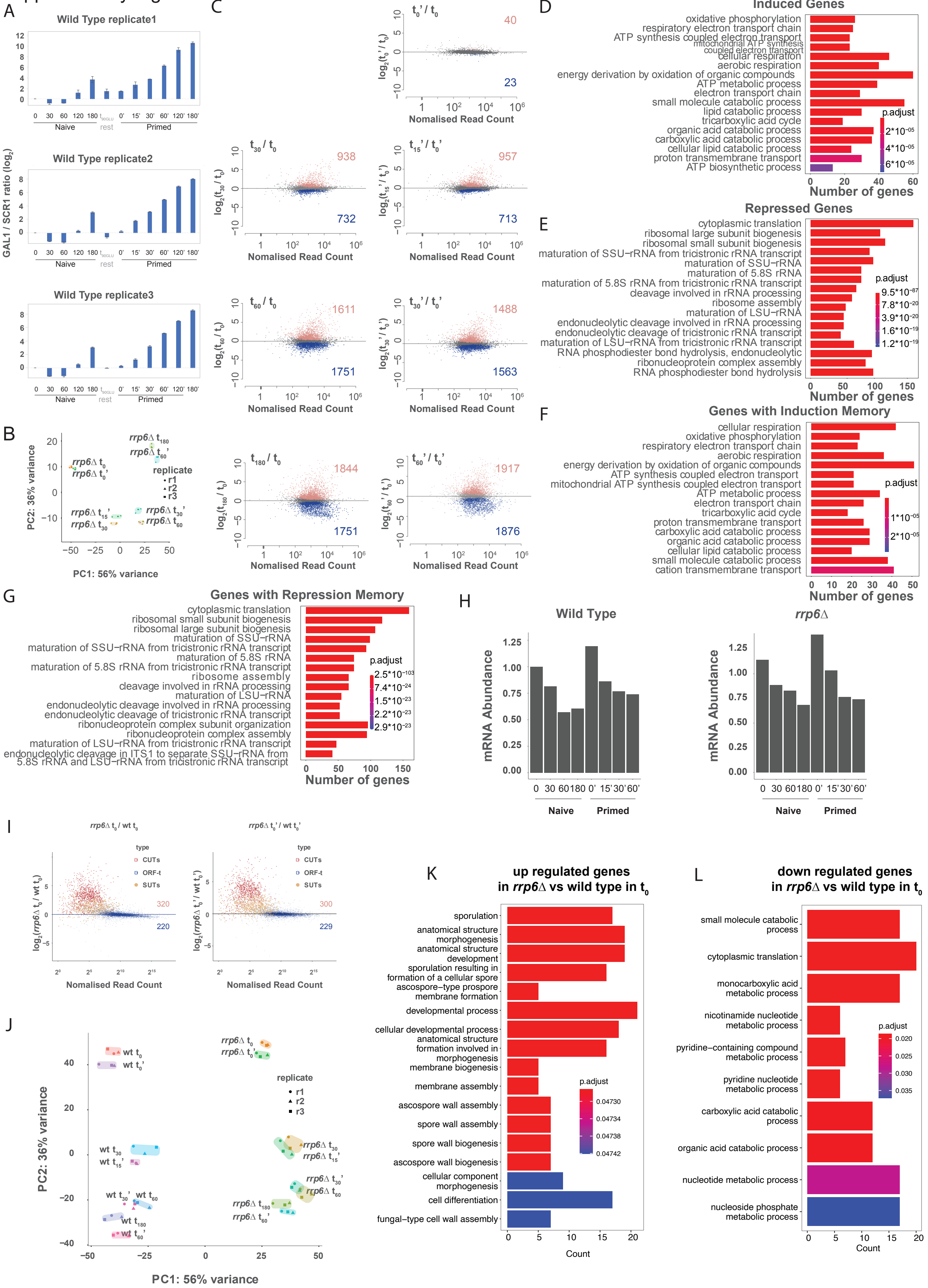

**Supplementary Figure 2: Differential regulation of mRNA abundance in naïve and prime states. A,** RT-qPCR analysis of the relative abundance of endogenous GAL1 mRNA normalised to SCR1 for both first and second galactose induction. Data are presented as mean values, error bars depict +/- SD of technical qPCR replicates. Independent biological replicates are shown in the 3 sub-panels. **B**, Principal component analysis (PCA) of mRNA expression (normalised RNA-Seq data) for both first and second induction in *rrp6Δ*. Only coding mRNAs (ORF-Ts) where considered. **C**, Differential coding gene expression across samples (ORF-Ts). MA plots show $log_2$ fold change differences on the y-axis and average normalised read counts (by global ORF-Ts abundance) on the x-axis. Time points in naive state are compared to time 0 (naive states). Time points in primed state are compared to time 0' (primed states). Significantly upregulated genes (p-adj < 0.001) are shown in red and significantly down-regulated (p-adj < 0.001) in blue. Wald test were used here and Benjamini-Hochberg adjustment were used for multiple comparations. Data comes from n=3 independent biological replicates. **D**, Gene Ontology enrichment analysis for induced genes in the wild-type strain. Only top GO listed, please see Supplementary Data S2 for the complete list. Over representation (hypergeometric test) analysis were used for enrichment test and Benjamini-Hochberg adjustment was used for multiple comparations. **E**, as D but for repressed genes. **F**, as D but for genes with induction memory. **G**, as D but for genes with repression memory. **H**, Total RNA abundance (normalised by spike in) of both first and second induction in wild-type and *rrp6Δ* strains. **I**, Differential coding gene expression across samples (ORF-Ts). MA plots show $log_2$ fold change differences on the y-axis and average normalised read counts (by global ORF-Ts abundance) on the x-axis. Time 0 of *rrp6Δ* in naive state are compared to time 0 of wild type (naive states). Time 0' of *rrp6Δ* in primed state are compared to time 0' of wild type (primed states). **J**, Principal component analysis (PCA) of mRNA expression (normalised RNA-Seq data) for both first and second induction in *rrp6Δ* as in Figure 2B, but including ORF-Ts, CUTs and SUTs. RNA abundance was normalized using coding genes. **K,** as D but for upregulated genes in *rrp6Δ*. **L,** as D but for downregulated genes in *rrp6Δ*.

# Supplementary Figure 3

A

**Promoter overlap with CUTs**

| | Gene Groups | total | expected | observed | p value | enrich | over |
|---|---|---|---|---|---|---|---|
| Induction Memory | **Attenuated** | 7 | 0.49 | 2 | 0.08 | over | 4.06 |
| | **not changed** | 451 | 31.71 | 30 | 0.42 | under | 1.06 |
| | **enhanced** | 88 | 6.19 | 10 | 0.09 | over | 1.62 |
| | Induced No Memory | 336 | 23.62 | 29 | 0.14 | over | 1.23 |
| | No Change | 3526 | 247.94 | 244 | 0.35 | under | 1.02 |
| | repressed No Memory | 294 | 20.67 | 23 | 0.33 | over | 1.11 |
| Repression Memory | **Attenuated** | 4 | 0.28 | 0 | 0.75 | under | 0 |
| | **not changed** | 611 | 42.96 | 35 | 0.10 | under | 1.23 |
| | **enhanced** | 158 | 11.11 | 12 | 0.43 | over | 1.08 |

B

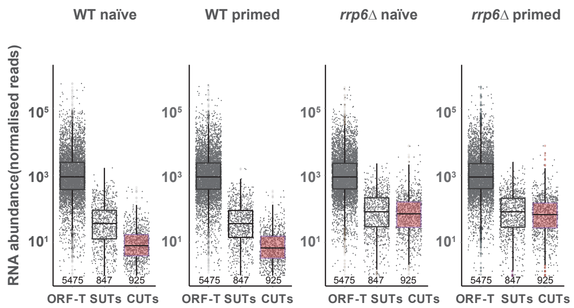


C

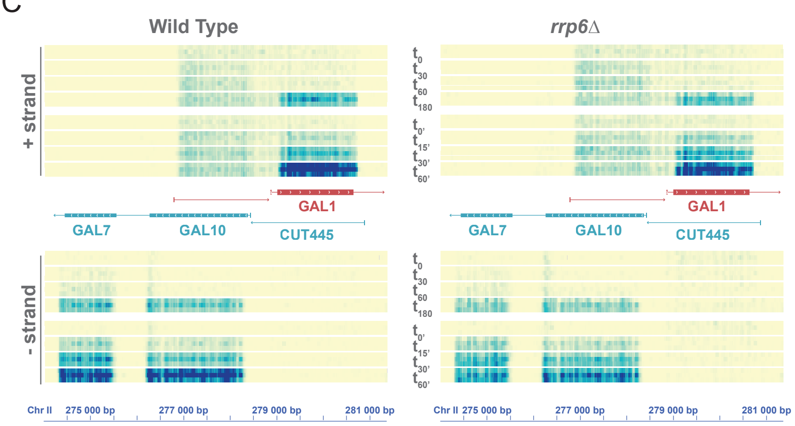


D

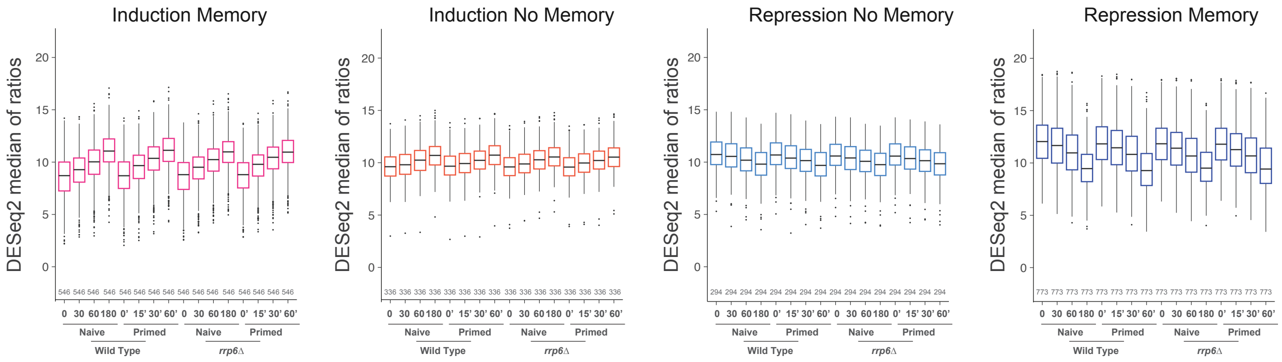


E

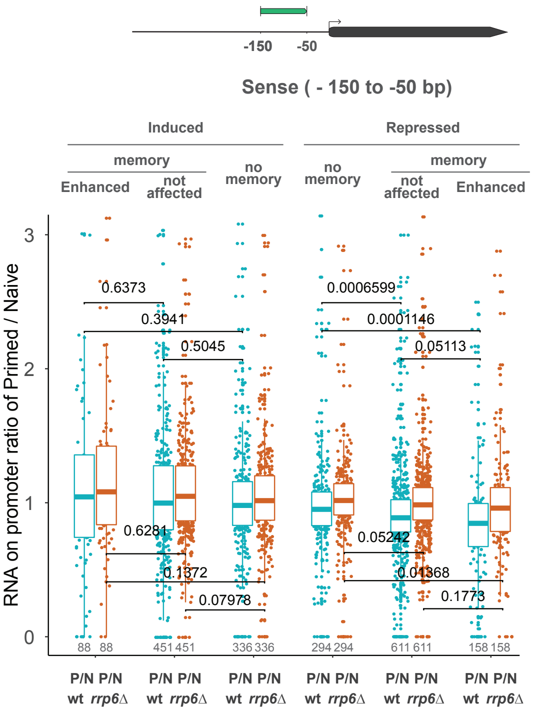


F

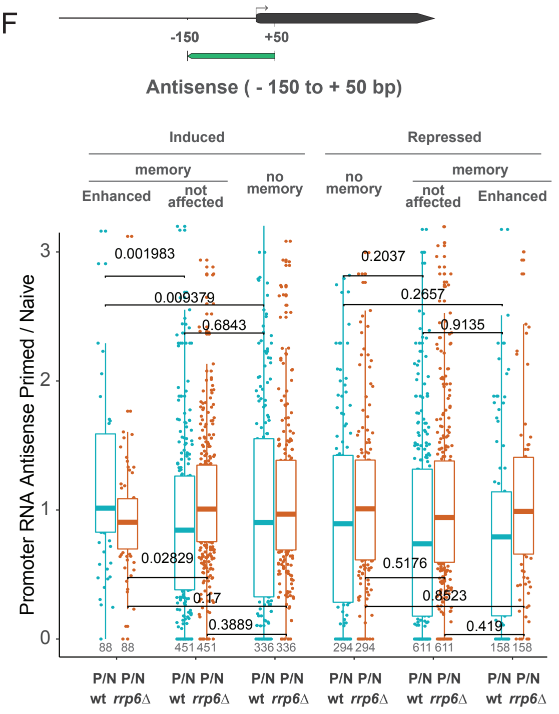

**Supplementary Figure 3: Contribution of transcription and chromatin organization to transcriptional memory.** **A**, Overlap between annotated CUTs and gene groups with different transcriptional memory profiles. Number of overlaps was computed between the transcription start sites and CUTs from[25]. (*i.e.* -150 nt to -50 relative to transcription start site on the sense strand and -150 to +50 on antisense strand). The significance for the overlap was tested using a hypergeometric test. Over representation analysis (hypergeometric test, one sided) was used and Benjamini-Hochberg adjustment were used for multiple comparations. **B**, Relative expression of ORF-Ts, SUTS and CUTs in naïve and primed cells for the wild type and the *rrp6*Δ strains. mRNA abundance is normalized to global ORF-Ts abundance. 5475 ORF-Ts, 847 SUTs and 925 CUTs examined over 3 independent biological experiments. First quantile, median and third quantile are defined as the minimum, center and maximum bounds of the box-plots. **C**, Strand-specific RNA-Seq coverage for the region containing *GAL1*, *GAL10* and *GAL7* in wild type and *rrp6*Δ strains (yellow low expressed to blue, high expressed). **D**, Box-plot for RNA abundance of studied genes (DESeq2's median of ratios). Box-plot represent the first, second and third quartile. 546 genes examined for induction memory, 336 for induction no memory, 294 for repression no memory and 773 for repression memory over 3 independent biological experiments. First quantile, median and third quantile are defined as the minimum, center and maximum bounds of the box-plots. **E**, RNA-Seq coverage ratio over the promoter region in sense strand (-150 bp to -50 bp) comparing primed vs naïve states (P/N) in wild-type (cyan) and *rrp6*Δ (brown). P value (two-sided Wilcoxon signed-rank test) refers to differences between gene groups within the same strain. For all boxplots the number of genes examined is depicted in the bottom of the boxplot and the independent biological experiments n= 3. First quantile, median and third quantile are defined as the minimum, center and maximum bounds of the box-plots. **F**, As in E but antisense covering the promoter region (-150 bp to +50 bp).

Supplementary Figure 4

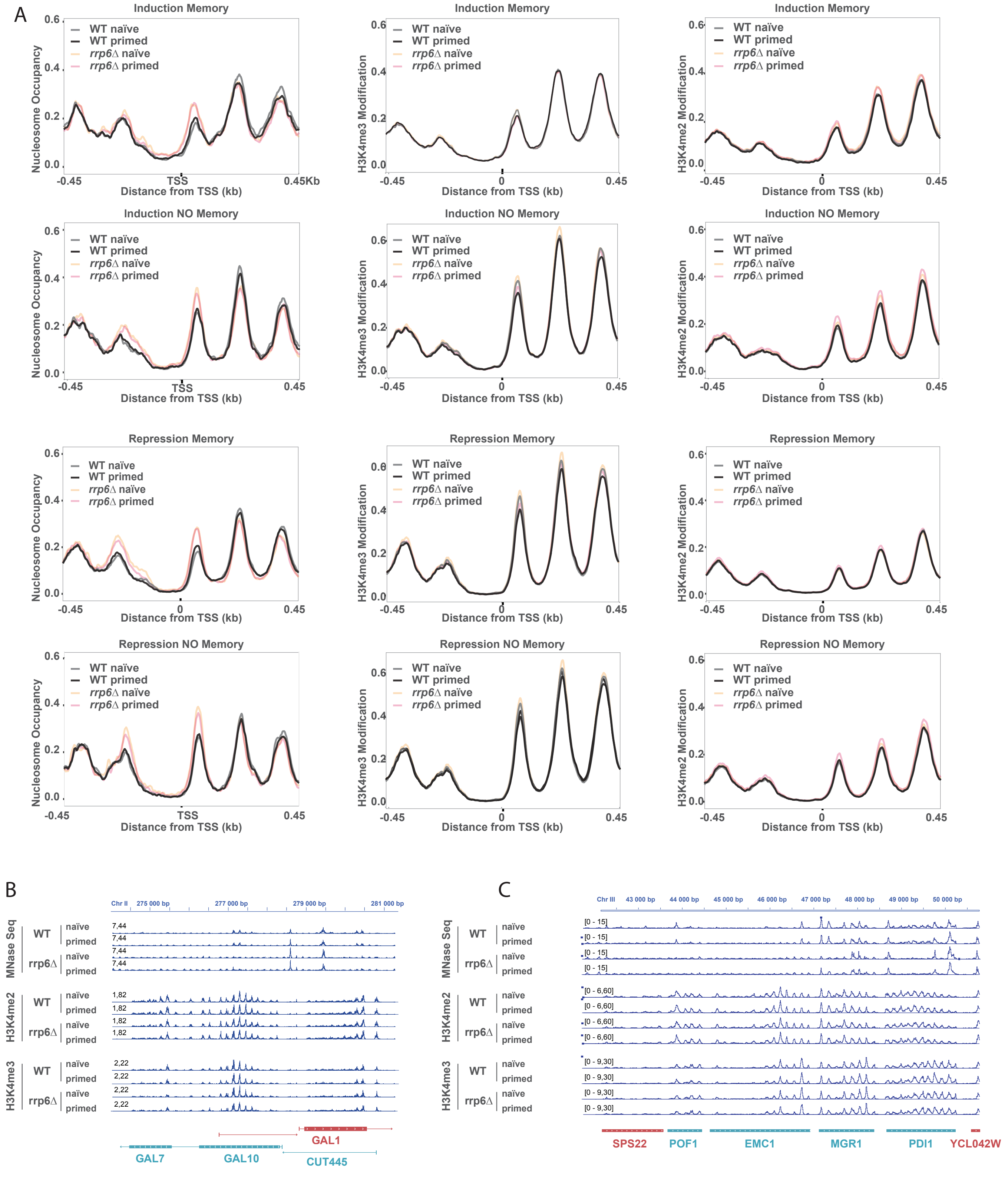


**Supplementary Figure 4: Contribution of chromatin organization to transcriptional memory.** A, Global MNase and ChIP Seq analysis. Metagene plot of the distribution of average nucleosome, H3K4me3, H3K4me2 signal. Average sequencing coverage is shown (cpm, counts per million) for wild-type $t_0$ naïve (grey), wild-type $t_0$' primed (black), *rrp6*Δ $t_0$ naïve (orange) and *rrp6*Δ $t_0$' primed (pink) here. Genome-wide chromatin profiles around transcription start sites (TSS). Average sequencing coverage is shown (cpm, counts per million) for nucleosome mapping (MNase, left column), H3K4me3 (center column) and H3K4me2 (right column). **B**, MNase-Seq and ChIP-Seq coverage for the region containing *GAL1*, *GAL10* and *GAL7* in wild type and *rrp6*Δ strains. Coverage in cpm, counts per million. **C**, as in B, but corresponding the region between SPS22 and PDI1 in Chromosome III.

Supplementary Figure 5

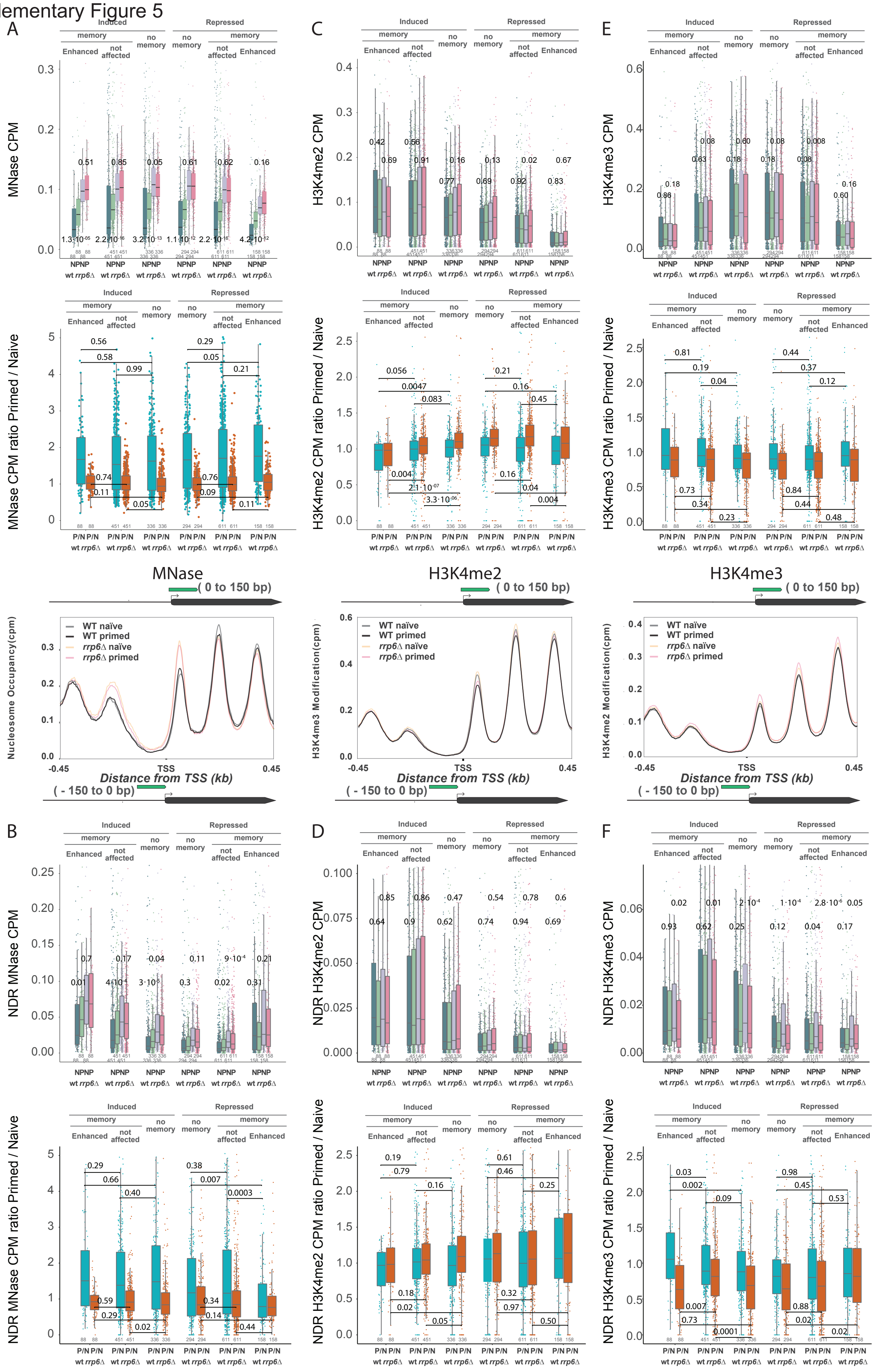

**Supplementary Figure 5: Contribution of Chromatin organization to transcriptional memory. A,** Upper part depicts the MNase Seq coverage over the nucleosome +1 (0 to 150bp from the TSS) for naïve and primed cells in the wild-type and the *rrp6*Δ strain. P value (Wilcoxon signed-rank test) refers to differences between gene groups within the same strain. Box-plot represent the first, second and third quartile. The bottom part depicts the MNase Seq coverage ratio over the nucleosome +1comparing primed vs naïve states in wild-type (cyan) and *rrp6*Δ (brown). For all boxplots the number of genes examined is depicted in the bottom of the boxplot and the independent biological experiments n= 3. First quantile, median and third quantile are defined as the minimum, center and maximum bounds of the box-plots. Two-sided Wilcoxon test was used for all comparisons. **B,** as A but depicting the MNAseq coverage for the Nucleosome Depleted Region (NDR), -150 to 0 bp from the TSS. **C-D,** as A-B but for H3K4me2. **E-F,** as A-B but for H3K4me3. Metagene from Figure 3B-D is shown as reference for the regions analyzed.

Supplementary Figure 6

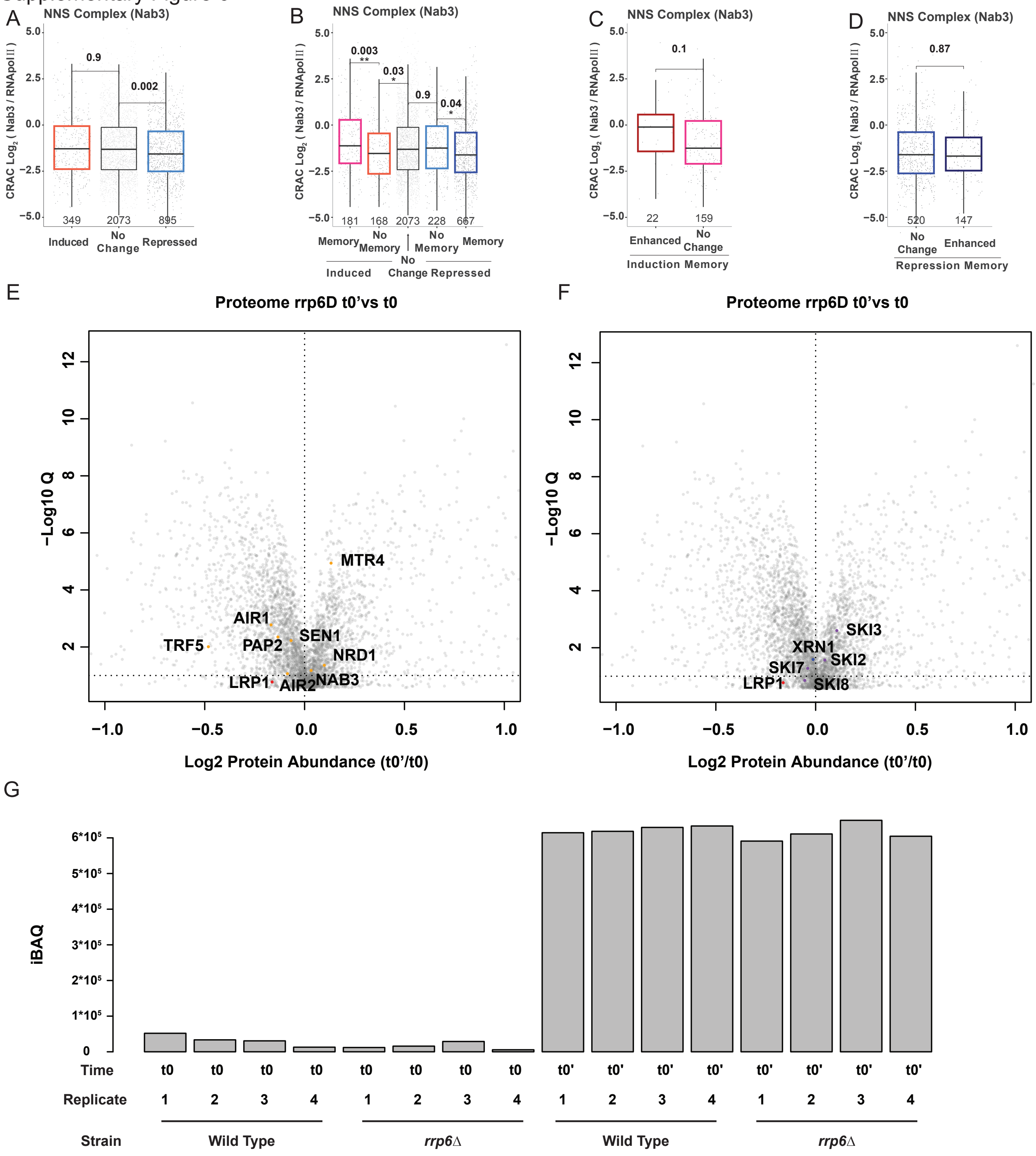


**Supplementary Figure 6: Differential association to NNS and remodelling of the proteome in primed conditions. A**, Relative association for the NNS complex (i.e. Nab3) as measured by CRAC in naïve cells [30]. Boxplot for induced and repressed genes is shown as in Figure 4. To measure gene-specific nuclear surveillance association, since those complexes act on nascent transcripts, CRAC data is normalized by RNA pol II association, as previously described. Number of analysed genes is indicated in grey. Genes with less than 20 counts were discarded. For all boxplots the number of genes examined is depicted in the bottom of the boxplot. First quantile, median and third quantile are defined as the minimum, center and maximum bounds of the box-plots. Two-sided Wilcoxon test was used for all comparisons. **B**, as A but for genes classified according to their transcriptional memory. **C**, As in A but comparing genes with induction memory enhanced or not enhanced by *RRP6* depletion. **D**, As in A but comparing genes with repression memory enhanced or not enhanced by RRP6 depletion. Significance computed using Wilcoxon signed-rank

test. CRAC data from Bresson *et al.*[30] **E**, Volcano plot showing relative protein abundance changes of primed ($t_{0'}$) and naïve ($t_0$) states in *rrp6*Δ cells. Fold changes in protein abundance for $t_{0'}$ and $t_0$ samples are shown as a function of statistical significance (n = 4 of independent replicates). The points showing the protein subunits of the TRAMP, NNS and nuclear exosome are labelled. **F**, same data shown in A with labels pinpointing components of the nuclear and cytoplasmic exosome and the 5'-3' exonuclease XRN1. **G**, GAL1 protein levels of naïve state and primed state in both wild type and rrp6Δ measured using intensity Based Absolute Quantification (iBAQ). Manual inspection of the raw MS spectra confirmed that GAL1 quantification in naïve conditions was below the limit of reliable detection.

Supplementary Figure 7

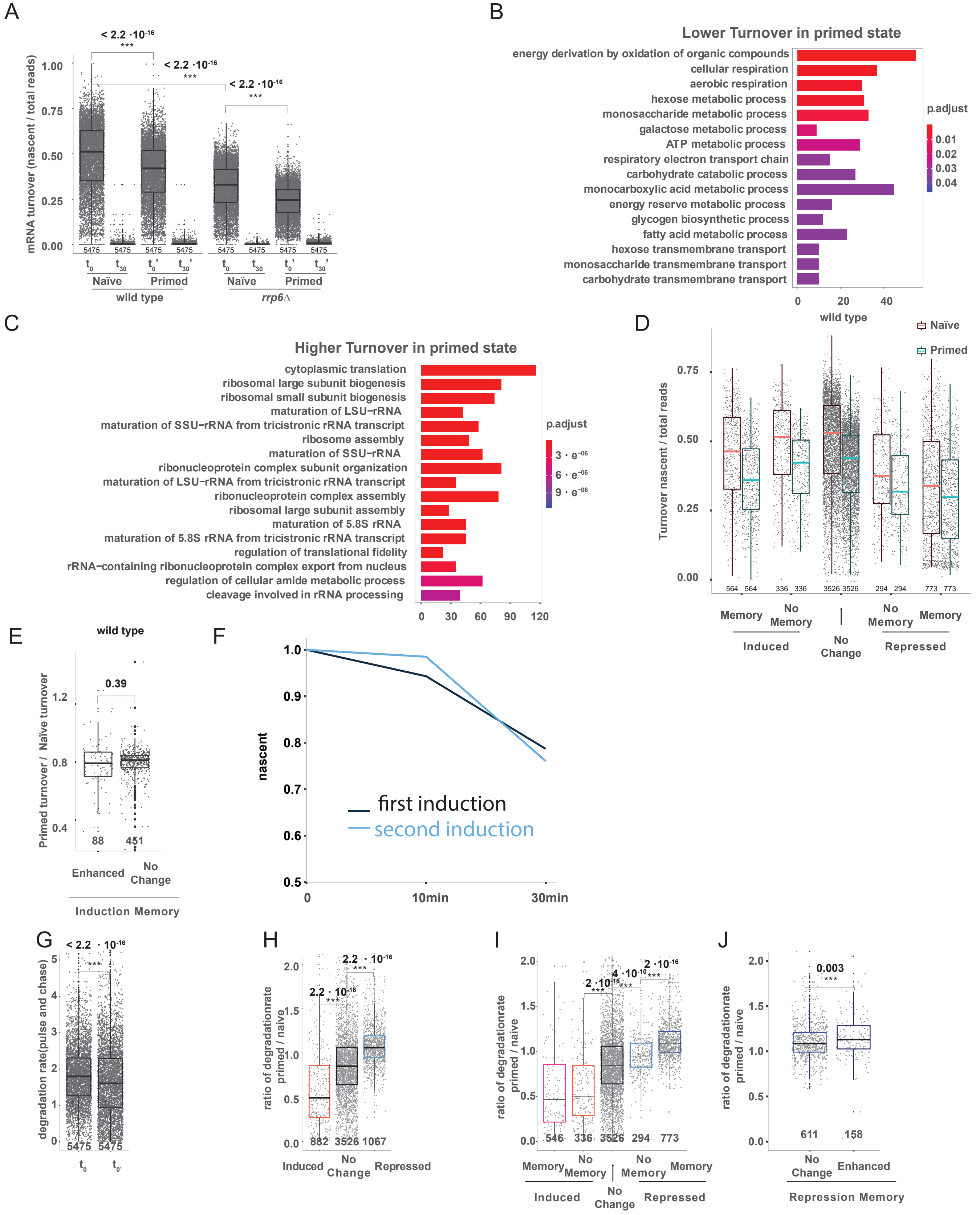


**Supplementary Figure 7: Expanded information for SLAM-Seq comparing naïve and primed states. A**, Relative mRNA turnover (comparing nascent vs total RNA) using SLAM-seq in naïve and primed conditions at $t_0$ and $t_{30}$ for the wild-type and the *rrp6Δ* strain. Independent biological experiments n= 3.**B-C,** Gene Ontology enrichment analysis for genes with relatively decreased (B) or increased (C) mRNA turnover in primed cells. Over representation analysis (hypergeometric test) were used for enrichment test and

Benjamini-Hochberg adjustment were used for multiple comparations. **D**, mRNA turnover in naïve ($t_0$) and primed ($t_0$') conditions according to memory gene classification in wild type strain. Independent biological experiments n= 3. **E**, in wild type strain, change in mRNA turnover between primed and naïve conditions for genes with induction memory enhanced or not enhanced by *RRP6* depletion. **F**, Genome-wide ratio of nascent (*4sU labelled*) vs total RNA for all coding genes using a pulse-chase approach in the wild-type strain. Ratio at time 0 was set to 1. **G**, Degradation rate (60 * ln(2) / t1/2) for all coding genes at time 0 for naïve (t0) and primed (t0') states in the wild-type strain (pulse-chase). **H**, ratio of degradation rate comparing primed vs naïve state for induced and repressed genes in the wild-type strain (pulse-chase). **I**, as G but for induction memory genes (pink), induced gene with no memory (orange), repressed genes with no memory (light blue) and repression memory genes (dark blue). **J**, as G but for genes with repression memory not affected or enhanced by RRP6 depletion. Box-plots represent the first, second and third quartile. For all boxplots the number of genes examined is depicted in the bottom of the boxplot. First quantile, median and third quantile are defined as the minimum, center and maximum bounds of the box-plots. When comparing box-plot distributions, two-sided Wilcoxon test was used.

Supplementary Figure 8

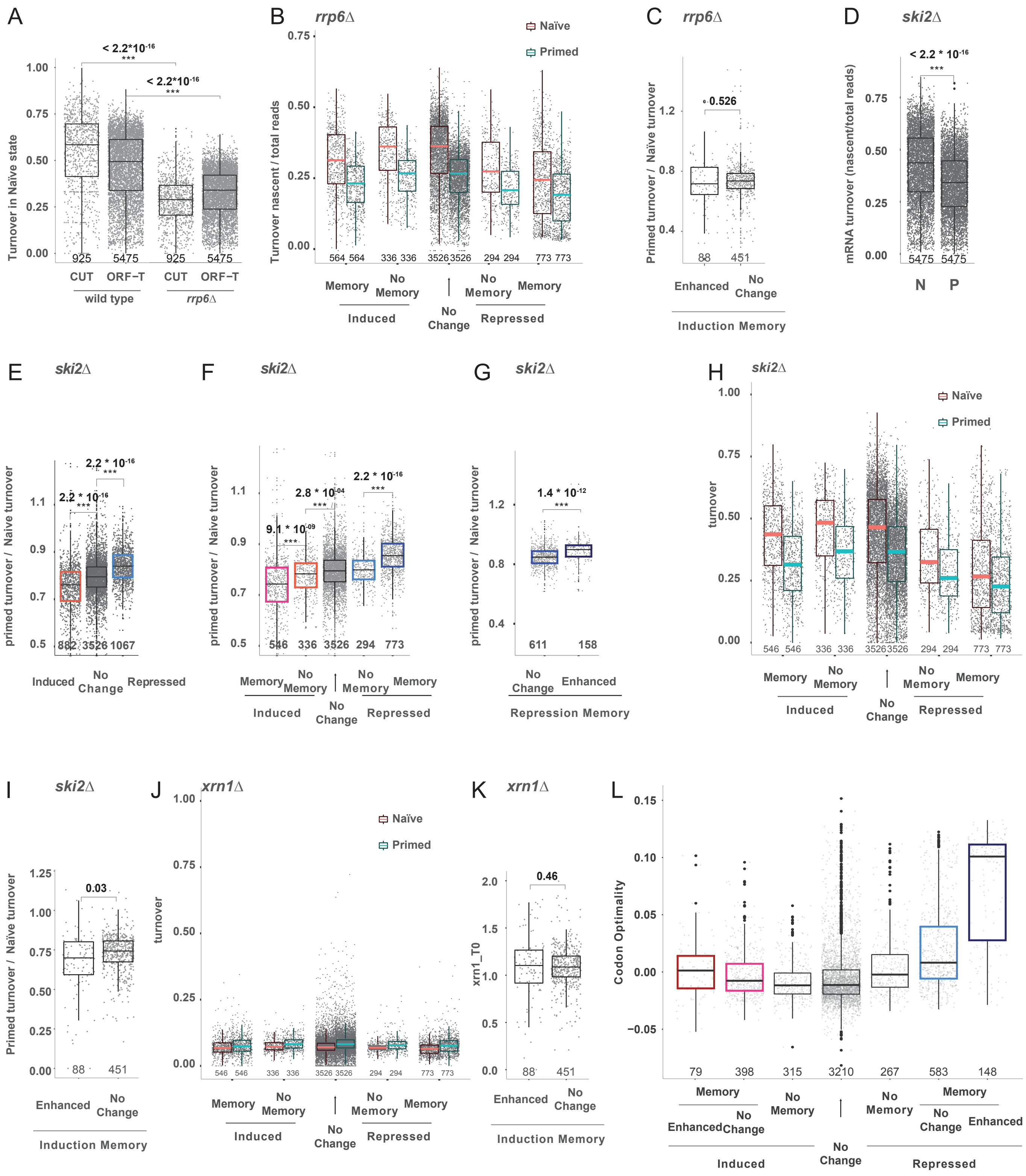


**Supplementary Figure 8: A**, Relative mRNA turnover (comparing nascent vs total RNA) in wild-type and *rrp6*Δ naïve $t_0$ state for CUTs and ORF-Ts. n= 5475 ORF-Ts and n = 925 CUTs examined over 3 independent biological experiments. **B**, mRNA turnover in naïve ($t_0$) and primed ($t_0$') conditions according to memory gene classification in *rrp6*Δ strain. **C**, same as B but comparing genes with *rrp6*Δ enhanced induction memory vs non enhanced induction memory. **D**, Relative mRNA turnover (comparing nascent vs total RNA) using SLAM-seq in naïve (N, $t_0$) and primed (P, $t_0$') conditions for the *ski2*Δ strain. Gene-specific turnover was computed as in Figure 5, comparing for each gene the reads containing T>C conversion to the total mapped reads. Only reads containing at least 2 T>C conversions are considered newly synthesized reads. Only genes with at least 20 total reads were considered for RNA turnover analysis. **E**, Change in mRNA turnover

between primed and naïve conditions for induced and repressed genes in response to galactose for the *ski2Δ* strain. **F**, As in D but for genes according to their transcriptional memory profile. **G**, As in D but for genes with repression memory enhanced or not enhanced after RRP6 depletion. **H,** same as B but for *ski2Δ*. **I,** same as B but but for *ski2Δ*. **J,** same as B but for *xrn1Δ*. **K,** same as B but for *xrn1Δ*. **L,** codon stability coefficients (CSCg) of gene groups (gene groups with less than 10 genes are omitted). Box-plot represent the first, second and third quartile. For all boxplots the number of genes examined is depicted in the bottom of the boxplot and derives from n03 independent biological replicates. First quantile, median and third quantile are defined as the minimum, center and maximum bounds of the box-plots. Two-sided Wilcoxon test was used for all comparisons.
