# Supplementary material for: Differential regulation of mRNA stability modulates transcriptional memory and facilitates environmental adaptation
Source: Nat Commun. 2023 Feb 17;14:910. doi: 10.1038/s41467-023-36586-x (PMC9936472; doi:10.1038/s41467-023-36586-x)
Supplement: Supplementary file 3 — Description of Additional Supplementary Files [file 41467_2023_36586_MOESM3_ESM.pdf]

Description of Additional Supplementary Files

File Name: Supplementary Data 1
Description: **Results of the transcriptional memory screen.** Microsoft Excel workbook containing the summary of cells sorted at each timepoint by FACS, the raw sequencing count for each time window and replicate, and the final list of candidates.

File Name: Supplementary Data 2
Description: **Generated RNA-Seq information.** S2A contains raw count, log_2 fold-change, gene type (ORF-T, CUTs or SUTs) and final gene category in column named gene groups. S2A Raw counts are direct output of featurecounts. S2A Log_2 fold change of each sample was compared to time 0 of naïve state in corresponding strain. S2A Gene group column contains nine gene groups: induction memory enhanced in *rrp6Δ*, induction memory that does not change, induction memory attenuated in *rrp6Δ,* genes induced without memory, genes with no significant change, genes repressed without memory, repression memory attenuated in *rrp6Δ*, repression memory not changed in *rrp6Δ* and repression memory enhanced in *rrp6Δ*. **S2B** Comparison of gene expression in naïve state between rrp6Δ and wild type. **S2C-S2H** Gene Ontology Enrichment for each gene group.

File Name: Supplementary Data 3
Description: **CRAC reanalisis. S3A** calculated CRAC data of Trf5 first normalised by total counts then normalised by RNApolII. **S3B** calculated CRAC data of Trf4 first normalised by total counts then normalised by RNApolII. **S3C** calculated CRAC data of Mtr4 first normalised by total counts then normalised by RNApolII. **S3D** calculated CRAC data of Ski2 first normalised by total counts then normalised by RNApolII. **S3E** calculated CRAC data of Nab3 first normalised by total counts then normalised by RNApolII. **S3F** calculated CRAC data of RNApolII normalised by total counts.

File Name: Supplementary Data 4
Description: **Proteomic differences between naïve and primed conditions.** Differential expression analysis for proteomic datasets. Four independent biological replicates where used. We compare wild-type naïve (BY-t0), wild-type primed (BY-t1), *rrp6Δ* naïve (rrp6-t0) and *rrp6Δ* primed (rrp6-t1).

File Name: Supplementary Data 5
Description: **Raw data for SLAM-seq analysis. S5A**: Nascent / Total ratio of each gene at time 0 and 30 min in naïve and primed states in both wild type and *rrp6Δ*. GO enrichment of genes which demonstrate relative lower turnover (**S5B**) and higher (**S5C**) turnover in primed state compared to naïve state in wild type. **S5D:** Nascent / Total ratio of each gene at time 0 in naïve and primed states in *ski2Δ*. **S5E:** Nascent / Total ratio of each gene at time 0 in naïve and primed states in *xrn1Δ*. **S5F:** spike in normalised nascent read counts in naïve state in wild type. **S5G:** spike in normalised nascent read counts in priemd state in wild type.

File Name: Supplementary Data 6
Description: **Reagents and Resourced table.**
