# Supplementary material for: Differential regulation of mRNA stability modulates transcriptional memory and facilitates environmental adaptation
Source: Nat Commun. 2023 Feb 17;14:910. doi: 10.1038/s41467-023-36586-x (PMC9936472; doi:10.1038/s41467-023-36586-x)
Supplement: Supplementary file 10 — Reporting Summary [file 41467_2023_36586_MOESM10_ESM.pdf]

nature portfolio

Corresponding author(s): Vicent Pelechano

Last updated by author(s): Jan 16, 2023

Reporting Summary

Nature Portfolio wishes to improve the reproducibility of the work that we publish. This form provides structure for consistency and transparency in reporting. For further information on Nature Portfolio policies, see our Editorial Policies and the Editorial Policy Checklist.

Statistics

For all statistical analyses, confirm that the following items are present in the figure legend, table legend, main text, or Methods section.

n/a	Confirmed	
☐	☒	The exact sample size (n) for each experimental group/condition, given as a discrete number and unit of measurement
☐	☒	A statement on whether measurements were taken from distinct samples or whether the same sample was measured repeatedly
☐	☒	The statistical test(s) used AND whether they are one- or two-sided<br>*Only common tests should be described solely by name; describe more complex techniques in the Methods section.*
☐	☒	A description of all covariates tested
☐	☒	A description of any assumptions or corrections, such as tests of normality and adjustment for multiple comparisons
☐	☒	A full description of the statistical parameters including central tendency (e.g. means) or other basic estimates (e.g. regression coefficient) AND variation (e.g. standard deviation) or associated estimates of uncertainty (e.g. confidence intervals)
☐	☒	For null hypothesis testing, the test statistic (e.g. F, t, r) with confidence intervals, effect sizes, degrees of freedom and P value noted<br>*Give P values as exact values whenever suitable.*
☒	☐	For Bayesian analysis, information on the choice of priors and Markov chain Monte Carlo settings
☒	☐	For hierarchical and complex designs, identification of the appropriate level for tests and full reporting of outcomes
☐	☒	Estimates of effect sizes (e.g. Cohen's d, Pearson's r), indicating how they were calculated

Our web collection on statistics for biologists contains articles on many of the points above.

Software and code

Policy information about availability of computer code

Data collection	Data was collected using NextSeq 500 default software.
Data analysis	Software vaersions:<br>R/3.6.3<br>deepTools/3.1.0<br>gnuparallel/20180822<br>ucsc-utilities/v345<br>sambamba/0.6.6<br>samtools/1.9<br>subread/1.5.2<br>MultiQC/1.7<br>DESeq2/1.26.0<br>ComplexHeatmap/2.2.0<br>tidyverse/1.3.0<br>Nextflow/20.10.0<br>nf-core/slamseq/1.0.0<br>TrimGalore/0.6.5<br>bwa/0.7.17<br>SeqKit/0.15.0<br>nf-core/mnaseseq/dev

ea-utils/1.1.2.537
Codes are available at : git@github.com:PelechanoLab/2022TranscriptionalMemoryLab.git

For manuscripts utilizing custom algorithms or software that are central to the research but not yet described in published literature, software must be made available to editors and reviewers. We strongly encourage code deposition in a community repository (e.g. GitHub). See the Nature Portfolio guidelines for submitting code & software for further information.

Data

Policy information about availability of data

All manuscripts must include a data availability statement. This statement should provide the following information, where applicable:

- Accession codes, unique identifiers, or web links for publicly available datasets
- A description of any restrictions on data availability
- For clinical datasets or third party data, please ensure that the statement adheres to our policy

Data generated in this study (ChIP-seq, RNA-seq, and MNase-seq) is deposited in GEO with accession numbers GSE201036 and GSE218400. The mass spectrometry proteomics data have been deposited to the ProteomeXchange Consortium via the PRIDE partner repository with the dataset identifier PXD036586. All bioinformatic analysis was performed as described in the methods section. No additional code was developed.

Human research participants

Policy information about studies involving human research participants and Sex and Gender in Research.

Reporting on sex and gender	No human research participants
Population characteristics	No human research participants
Recruitment	No human research participants
Ethics oversight	No human research participants

Note that full information on the approval of the study protocol must also be provided in the manuscript.

Field-specific reporting

Please select the one below that is the best fit for your research. If you are not sure, read the appropriate sections before making your selection.

☒ Life sciences ☐ Behavioural & social sciences ☐ Ecological, evolutionary & environmental sciences

For a reference copy of the document with all sections, see nature.com/documents/nr-reporting-summary-flat.pdf

Life sciences study design

All studies must disclose on these points even when the disclosure is negative.

Sample size	We first response to Galactose in naive and primed S. cerevisiae cells. We performed RNA-Seq, SLAM-seq (RNA turnover), MNase-Seq, ChiP-Seq and Proteomic analysis in multiple wild type and mutant strains for components of the RNA degradation machinery. No sample size calculation was performed. Due to the relatively low variability between biological replicates, triplicate experiments where judged as sufficient. Additionally, the variability across biological samples was considered when computing differential gene expression as is commonly performed in the field.
Data exclusions	In general we excluded the genes with less than 20 counts across all samples.
Replication	In general, each experiment was repeated 3 times using independent biological replicates. We confirm all attempts at replication were successful.
Randomization	Experiments were performed containing always wild type and mutants in the same experimental batch (to minimize potential batch effects confounding biological differences). As the number of samples analyzed was not big, samples were analized in the same sequencing run. Thus, there was no need for additional randomization.
Blinding	None. As is common practice in molecular biology experiments using wild-type and mutant strains blinding was not considered necessary. Additionally, the bioinformatic readout and initial clustering (e.g. Fig2b) was performed without knowing the identity of the samples a priory. This confirmed that the experiment was succesful.

Reporting for specific materials, systems and methods

We require information from authors about some types of materials, experimental systems and methods used in many studies. Here, indicate whether each material, system or method listed is relevant to your study. If you are not sure if a list item applies to your research, read the appropriate section before selecting a response.

Materials & experimental systems

n/a	Involved in the study
☐	☒ Antibodies
☒	☐ Eukaryotic cell lines
☒	☐ Palaeontology and archaeology
☒	☐ Animals and other organisms
☒	☐ Clinical data
☒	☐ Dual use research of concern

Methods

n/a	Involved in the study
☐	☒ ChIP-seq
☒	☐ Flow cytometry
☒	☐ MRI-based neuroimaging

Antibodies

Antibodies used

anti Histone H3 antibody(Active Motif 39763), anti H3K4me3 antibody(Abcam ab8580), anti H3K4me2 antibody(Abcam ab7766)

Validation

H3 Antibody:
Applications Validated by manufacturer Active Motif: Validation includes ChIP-Seq, ChIP, Immunofluorescence and western blot.
ChIP-Seq: 4 µg per ChIP

H3K4me3 and H3K4me2 Antibody :
Success from the first experiment – confirmed specificity through extensive validation by manufacturer Abcam.
Validation includes ChIP, Immunofluorescence, western blot and the use of a peptide array.

ChIP-seq

Data deposition

☒ Confirm that both raw and final processed data have been deposited in a public database such as GEO.

☒ Confirm that you have deposited or provided access to graph files (e.g. BED files) for the called peaks.

Data access links
May remain private before publication.

GSE201036

Files in database submission

ChIPseq_H3K4me2_WT_t1_rep1_S25_R1_001.fastq.gz
ChIPseq_H3K4me2_WT_t1_rep2_S26_R1_001.fastq.gz
ChIPseq_H3K4me2_WT_t1_rep3_S27_R1_001.fastq.gz
ChIPseq_H3K4me2_WT_t2_rep1_S31_R1_001.fastq.gz
ChIPseq_H3K4me2_WT_t2_rep2_S32_R1_001.fastq.gz
ChIPseq_H3K4me2_WT_t2_rep3_S33_R1_001.fastq.gz
ChIPseq_H3K4me2_rrp6_t1_rep1_S28_R1_001.fastq.gz
ChIPseq_H3K4me2_rrp6_t1_rep2_S29_R1_001.fastq.gz
ChIPseq_H3K4me2_rrp6_t1_rep3_S30_R1_001.fastq.gz
ChIPseq_H3K4me2_rrp6_t2_rep1_S34_R1_001.fastq.gz
ChIPseq_H3K4me2_rrp6_t2_rep2_S35_R1_001.fastq.gz
ChIPseq_H3K4me2_rrp6_t2_rep3_S36_R1_001.fastq.gz
ChIPseq_H3K4me3_WT_t1_rep1_S37_R1_001.fastq.gz
ChIPseq_H3K4me3_WT_t1_rep2_S38_R1_001.fastq.gz
ChIPseq_H3K4me3_WT_t1_rep3_S39_R1_001.fastq.gz
ChIPseq_H3K4me3_WT_t2_rep1_S43_R1_001.fastq.gz
ChIPseq_H3K4me3_WT_t2_rep2_S44_R1_001.fastq.gz
ChIPseq_H3K4me3_WT_t2_rep3_S45_R1_001.fastq.gz
ChIPseq_H3K4me3_rrp6_t1_rep1_S40_R1_001.fastq.gz
ChIPseq_H3K4me3_rrp6_t1_rep2_S41_R1_001.fastq.gz
ChIPseq_H3K4me3_rrp6_t1_rep3_S42_R1_001.fastq.gz
ChIPseq_H3K4me3_rrp6_t2_rep1_S46_R1_001.fastq.gz
ChIPseq_H3K4me3_rrp6_t2_rep2_S47_R1_001.fastq.gz
ChIPseq_H3K4me3_rrp6_t2_rep3_S48_R1_001.fastq.gz
ChIPseq_H3_WT_t1_rep1_S13_R1_001.fastq.gz
ChIPseq_H3_WT_t1_rep2_S14_R1_001.fastq.gz
ChIPseq_H3_WT_t1_rep3_S15_R1_001.fastq.gz
ChIPseq_H3_WT_t2_rep1_S19_R1_001.fastq.gz
ChIPseq_H3_WT_t2_rep2_S20_R1_001.fastq.gz
ChIPseq_H3_WT_t2_rep3_S21_R1_001.fastq.gz
ChIPseq_H3_rrp6_t1_rep1_S16_R1_001.fastq.gz
ChIPseq_H3_rrp6_t1_rep2_S17_R1_001.fastq.gz
ChIPseq_H3_rrp6_t1_rep3_S18_R1_001.fastq.gz

ChIPseq_H3_rrp6_t2_rep1_S22_R1_001.fastq.gz
ChIPseq_H3_rrp6_t2_rep2_S23_R1_001.fastq.gz
ChIPseq_H3_rrp6_t2_rep3_S24_R1_001.fastq.gz
ChIPseq_Input_WT_t1_rep1_S1_R1_001.fastq.gz
ChIPseq_Input_WT_t1_rep2_S2_R1_001.fastq.gz
ChIPseq_Input_WT_t1_rep3_S3_R1_001.fastq.gz
ChIPseq_Input_WT_t2_rep1_S7_R1_001.fastq.gz
ChIPseq_Input_WT_t2_rep2_S8_R1_001.fastq.gz
ChIPseq_Input_WT_t2_rep3_S9_R1_001.fastq.gz
H3K4me2_WT_t1_R1.mLb.clN.sorted.bw
H3K4me2_WT_t1_R2.mLb.clN.sorted.bw
H3K4me2_WT_t1_R3.mLb.clN.sorted.bw
H3K4me2_WT_t2_R1.mLb.clN.sorted.bw
H3K4me2_WT_t2_R2.mLb.clN.sorted.bw
H3K4me2_WT_t2_R3.mLb.clN.sorted.bw
H3K4me2_rrp6_t1_R1.mLb.clN.sorted.bw
H3K4me2_rrp6_t1_R2.mLb.clN.sorted.bw
H3K4me2_rrp6_t1_R3.mLb.clN.sorted.bw
H3K4me2_rrp6_t2_R1.mLb.clN.sorted.bw
H3K4me2_rrp6_t2_R2.mLb.clN.sorted.bw
H3K4me2_rrp6_t2_R3.mLb.clN.sorted.bw
H3K4me3_WT_t1_R1.mLb.clN.sorted.bw
H3K4me3_WT_t1_R2.mLb.clN.sorted.bw
H3K4me3_WT_t1_R3.mLb.clN.sorted.bw
H3K4me3_WT_t2_R1.mLb.clN.sorted.bw
H3K4me3_WT_t2_R2.mLb.clN.sorted.bw
H3K4me3_WT_t2_R3.mLb.clN.sorted.bw
H3K4me3_rrp6_t1_R1.mLb.clN.sorted.bw
H3K4me3_rrp6_t1_R2.mLb.clN.sorted.bw
H3K4me3_rrp6_t1_R3.mLb.clN.sorted.bw
H3K4me3_rrp6_t2_R1.mLb.clN.sorted.bw
H3K4me3_rrp6_t2_R2.mLb.clN.sorted.bw
H3K4me3_rrp6_t2_R3.mLb.clN.sorted.bw
H3_WT_t1_R1.mLb.clN.sorted.bw
H3_WT_t1_R2.mLb.clN.sorted.bw
H3_WT_t1_R3.mLb.clN.sorted.bw
H3_WT_t2_R1.mLb.clN.sorted.bw
H3_WT_t2_R2.mLb.clN.sorted.bw
H3_WT_t2_R3.mLb.clN.sorted.bw
H3_rrp6_t1_R1.mLb.clN.sorted.bw
H3_rrp6_t1_R2.mLb.clN.sorted.bw
H3_rrp6_t1_R3.mLb.clN.sorted.bw
H3_rrp6_t2_R1.mLb.clN.sorted.bw
H3_rrp6_t2_R2.mLb.clN.sorted.bw
H3_rrp6_t2_R3.mLb.clN.sorted.bw
Input_WT_t1_R1.mLb.clN.sorted.bw
Input_WT_t1_R2.mLb.clN.sorted.bw
Input_WT_t1_R3.mLb.clN.sorted.bw
Input_WT_t2_R1.mLb.clN.sorted.bw
Input_WT_t2_R2.mLb.clN.sorted.bw
Input_WT_t2_R3.mLb.clN.sorted.bw
Input_rrp6_t1_R1.mLb.clN.sorted.bw
Input_rrp6_t1_R2.mLb.clN.sorted.bw
Input_rrp6_t1_R3.mLb.clN.sorted.bw
Input_rrp6_t2_R1.mLb.clN.sorted.bw
Input_rrp6_t2_R2.mLb.clN.sorted.bw
Input_rrp6_t2_R3.mLb.clN.sorted.bw
ChIPseq_Input_rrp6_t1_rep1_S4_R1_001.fastq.gz
ChIPseq_Input_rrp6_t1_rep2_S5_R1_001.fastq.gz
ChIPseq_Input_rrp6_t1_rep3_S6_R1_001.fastq.gz
ChIPseq_Input_rrp6_t2_rep1_S10_R1_001.fastq.gz
ChIPseq_Input_rrp6_t2_rep2_S11_R1_001.fastq.gz
ChIPseq_Input_rrp6_t2_rep3_S12_R1_001.fastq.gz
ChIPseq_H3K4me2_WT_t1_rep1_S25_R2_001.fastq.gz
ChIPseq_H3K4me2_WT_t1_rep2_S26_R2_001.fastq.gz
ChIPseq_H3K4me2_WT_t1_rep3_S27_R2_001.fastq.gz
ChIPseq_H3K4me2_WT_t2_rep1_S31_R2_001.fastq.gz
ChIPseq_H3K4me2_WT_t2_rep2_S32_R2_001.fastq.gz
ChIPseq_H3K4me2_WT_t2_rep3_S33_R2_001.fastq.gz
ChIPseq_H3K4me2_rrp6_t1_rep1_S28_R2_001.fastq.gz
ChIPseq_H3K4me2_rrp6_t1_rep2_S29_R2_001.fastq.gz
ChIPseq_H3K4me2_rrp6_t1_rep3_S30_R2_001.fastq.gz
ChIPseq_H3K4me2_rrp6_t2_rep1_S34_R2_001.fastq.gz
ChIPseq_H3K4me2_rrp6_t2_rep2_S35_R2_001.fastq.gz
ChIPseq_H3K4me2_rrp6_t2_rep3_S36_R2_001.fastq.gz
ChIPseq_H3K4me3_WT_t1_rep1_S37_R2_001.fastq.gz

ChIPseq_H3K4me3_WT_t1_rep2_S38_R2_001.fastq.gz
ChIPseq_H3K4me3_WT_t1_rep3_S39_R2_001.fastq.gz
ChIPseq_H3K4me3_WT_t2_rep1_S43_R2_001.fastq.gz
ChIPseq_H3K4me3_WT_t2_rep2_S44_R2_001.fastq.gz
ChIPseq_H3K4me3_WT_t2_rep3_S45_R2_001.fastq.gz
ChIPseq_H3K4me3_rrp6_t1_rep1_S40_R2_001.fastq.gz
ChIPseq_H3K4me3_rrp6_t1_rep2_S41_R2_001.fastq.gz
ChIPseq_H3K4me3_rrp6_t1_rep3_S42_R2_001.fastq.gz
ChIPseq_H3K4me3_rrp6_t2_rep1_S46_R2_001.fastq.gz
ChIPseq_H3K4me3_rrp6_t2_rep2_S47_R2_001.fastq.gz
ChIPseq_H3K4me3_rrp6_t2_rep3_S48_R2_001.fastq.gz
ChIPseq_H3_WT_t1_rep1_S13_R2_001.fastq.gz
ChIPseq_H3_WT_t1_rep2_S14_R2_001.fastq.gz
ChIPseq_H3_WT_t1_rep3_S15_R2_001.fastq.gz
ChIPseq_H3_WT_t2_rep1_S19_R2_001.fastq.gz
ChIPseq_H3_WT_t2_rep2_S20_R2_001.fastq.gz
ChIPseq_H3_WT_t2_rep3_S21_R2_001.fastq.gz
ChIPseq_H3_rrp6_t1_rep1_S16_R2_001.fastq.gz
ChIPseq_H3_rrp6_t1_rep2_S17_R2_001.fastq.gz
ChIPseq_H3_rrp6_t1_rep3_S18_R2_001.fastq.gz
ChIPseq_H3_rrp6_t2_rep1_S22_R2_001.fastq.gz
ChIPseq_H3_rrp6_t2_rep2_S23_R2_001.fastq.gz
ChIPseq_H3_rrp6_t2_rep3_S24_R2_001.fastq.gz
ChIPseq_Input_WT_t1_rep1_S1_R2_001.fastq.gz
ChIPseq_Input_WT_t1_rep2_S2_R2_001.fastq.gz
ChIPseq_Input_WT_t1_rep3_S3_R2_001.fastq.gz
ChIPseq_Input_WT_t2_rep1_S7_R2_001.fastq.gz
ChIPseq_Input_WT_t2_rep2_S8_R2_001.fastq.gz
ChIPseq_Input_WT_t2_rep3_S9_R2_001.fastq.gz
ChIPseq_Input_rrp6_t1_rep1_S4_R2_001.fastq.gz
ChIPseq_Input_rrp6_t1_rep2_S5_R2_001.fastq.gz
ChIPseq_Input_rrp6_t1_rep3_S6_R2_001.fastq.gz
ChIPseq_Input_rrp6_t2_rep1_S10_R2_001.fastq.gz
ChIPseq_Input_rrp6_t2_rep2_S11_R2_001.fastq.gz
ChIPseq_Input_rrp6_t2_rep3_S12_R2_001.fastq.gz

Genome browser session
(e.g. UCSC)

Assembly: V64-1-1

Methodology

Replicates

each sample has three replicates

Sequencing depth

ChIP-Seq and MNaseq coverage 5.5-18.3M mapped reads. RNA-Seq 17.7-60.6M and SLAM-Seq 14.28-23.48M mapped reads. Additional details in GEO.

Antibodies

anti Histone H3 antibody(Active Motif 39763), anti H3K4me3 antibody(Abcam ab8580), anti H3K4me2 antibody(Abcam ab7766)

Peak calling parameters

Peak calling was not performed.

Data quality

FatsQC analysis. Exploration of data using IGV, and metagene and gene specific analysis confirming known biology of the investigated marks.

Software

R/3.6.3
deepTools/3.1.0
gnuparallel/20180822
ucsc-utilities/v345
sambamba/0.6.6
samtools/1.9
subread/1.5.2
MultiQC/1.7
DESeq2/1.26.0
ComplexHeatmap/2.2.0
tidyverse/1.3.0
Nextflow/20.10.0
nf-core/slamseq/1.0.0
TrimGalore/0.6.5
bwa/0.7.17
SeqKit/0.15.0
nf-core/mnaseseq/dev
ea-utils/1.1.2.537
